# Supplementary material for: Role of the Egr2 Promoter Antisense RNA in Modulating the Schwann Cell Chromatin Landscape
Source: Biomedicines. 2024 Nov 13;12(11):2594. doi: 10.3390/biomedicines12112594 (PMC11592338; doi:10.3390/biomedicines12112594)

Supplementary Figure S1 (Figure S1)

A

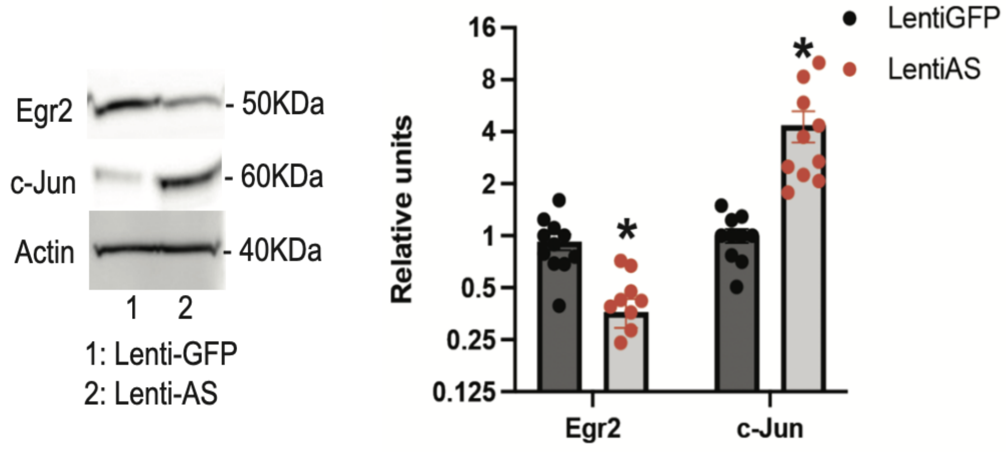

B

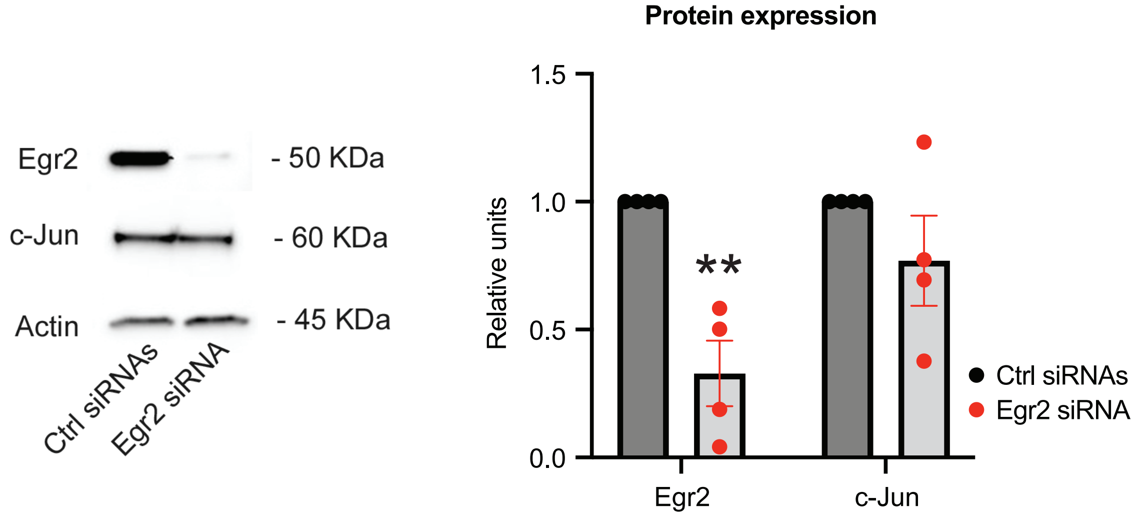

C

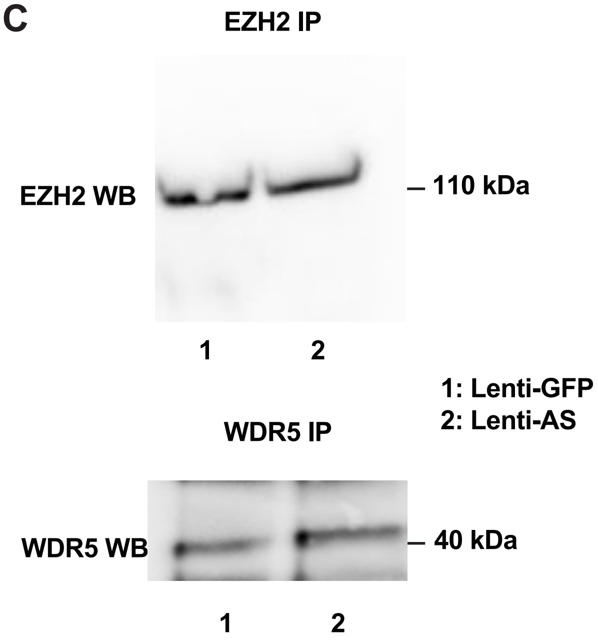

D

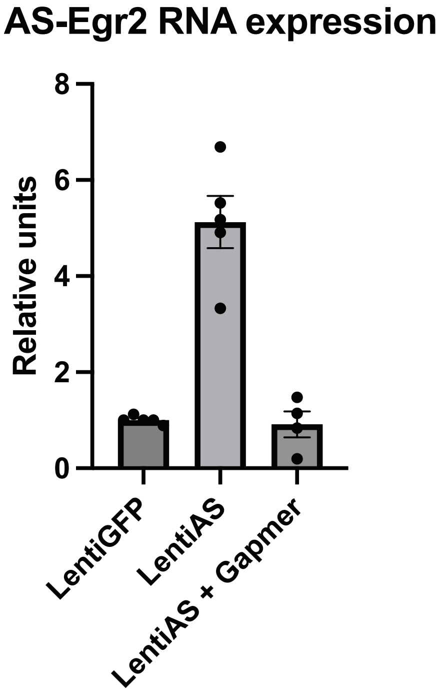

Supplementary Figure S2 (Figure S2)

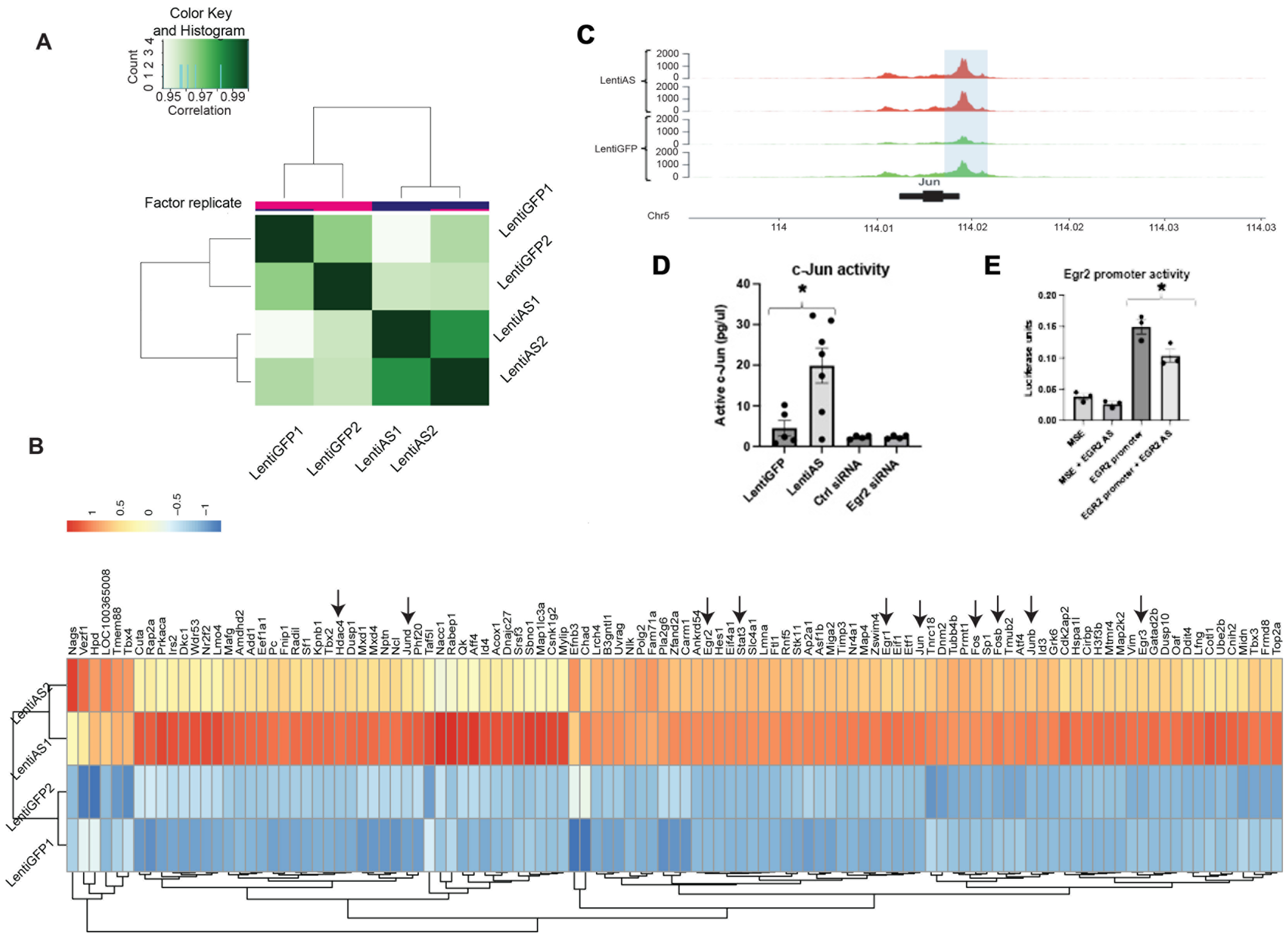

Supplementary Figure S3 (Figure S3)

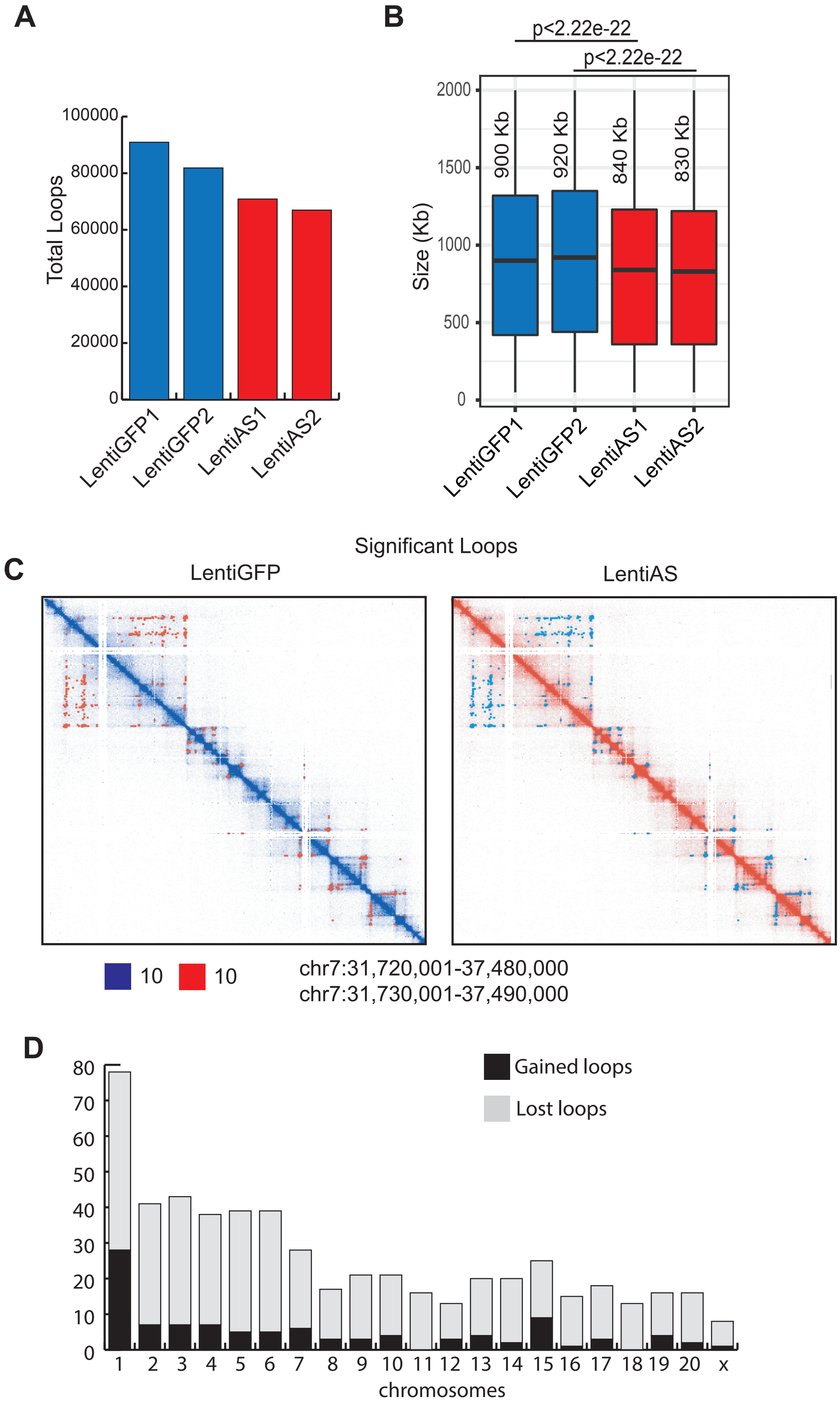

Supplementary Figure S4 (Figure S4)

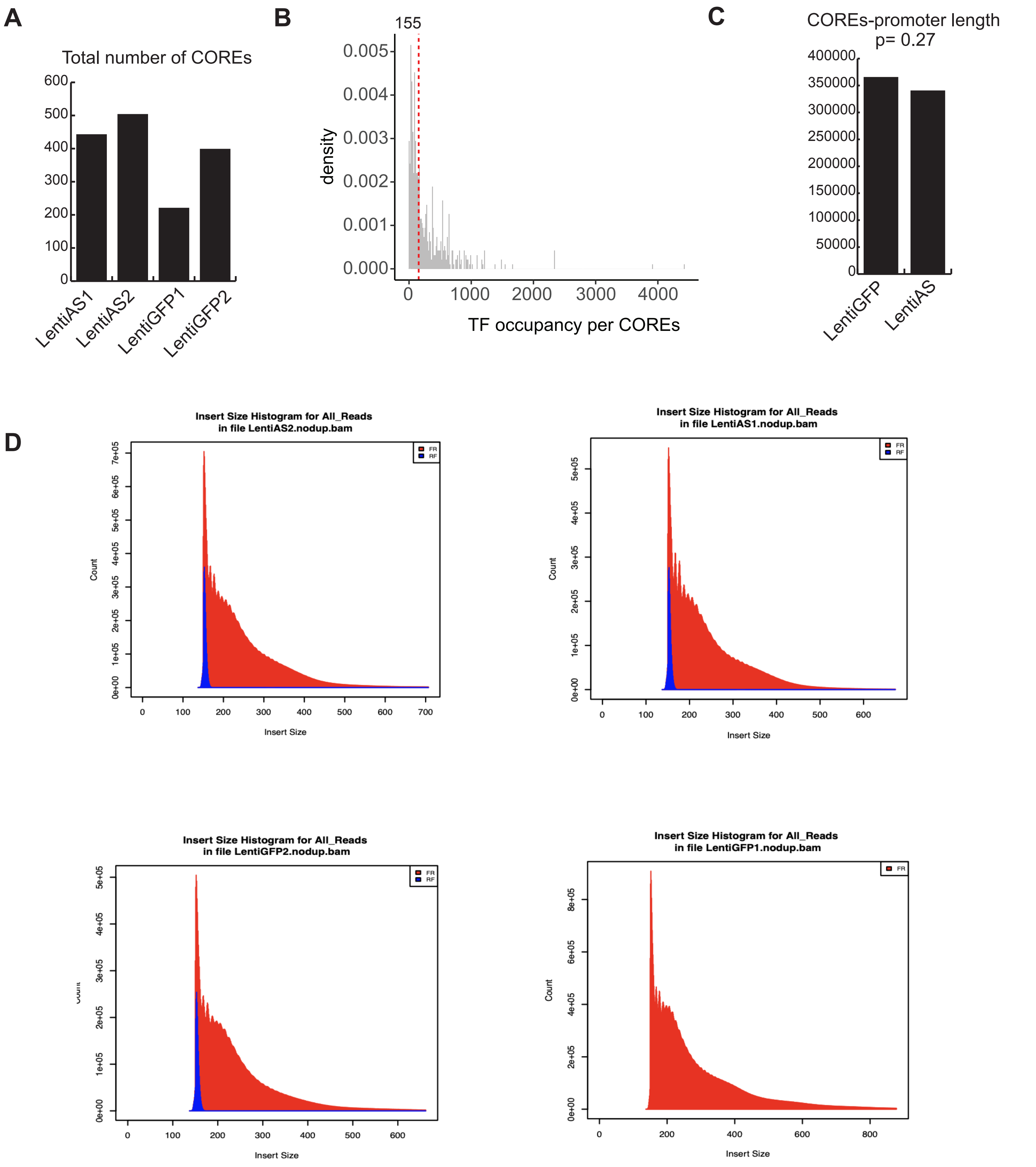

Supplementary Figure S5 (Figure S5)

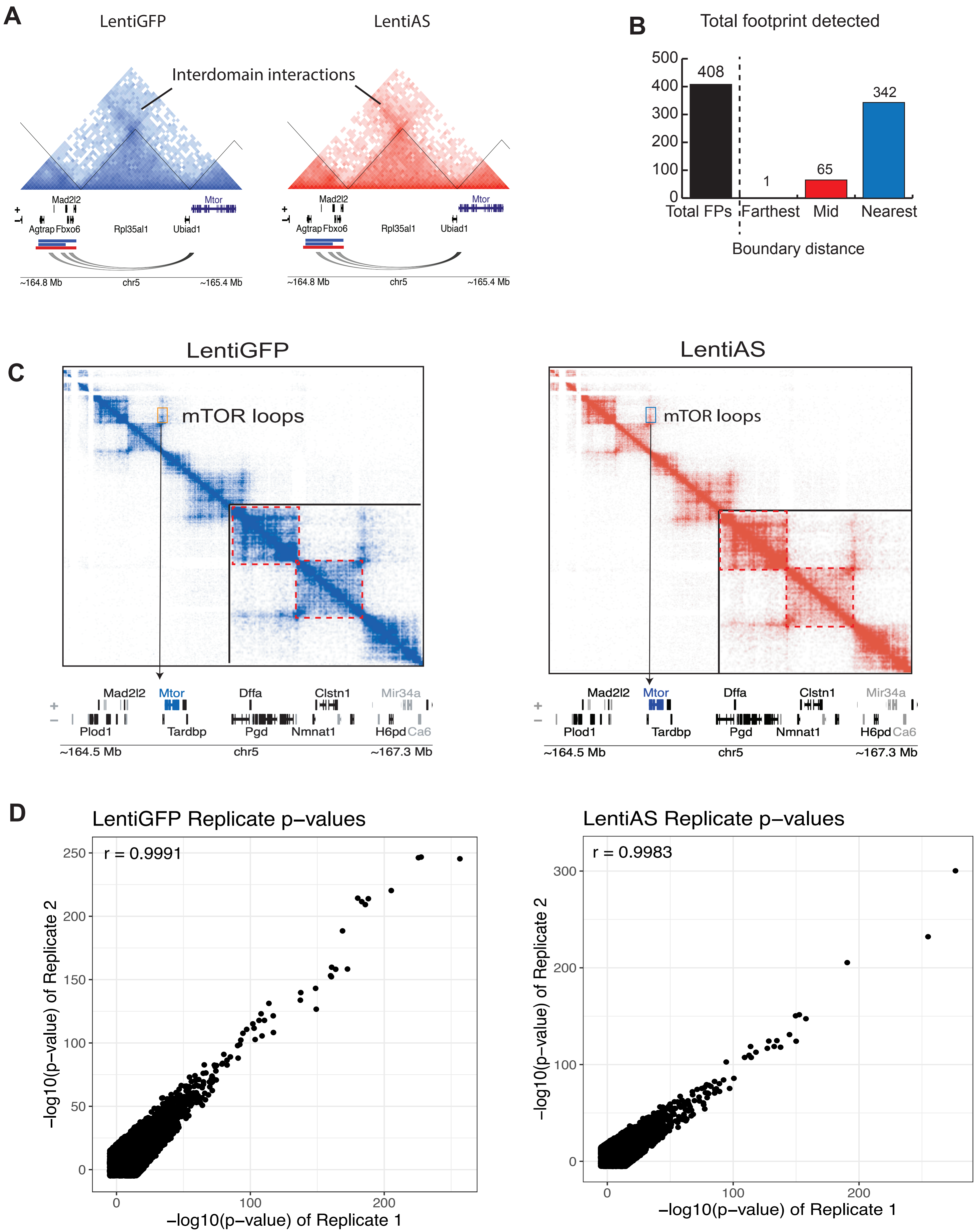

Supplementary Figure S6 (Figure S6)

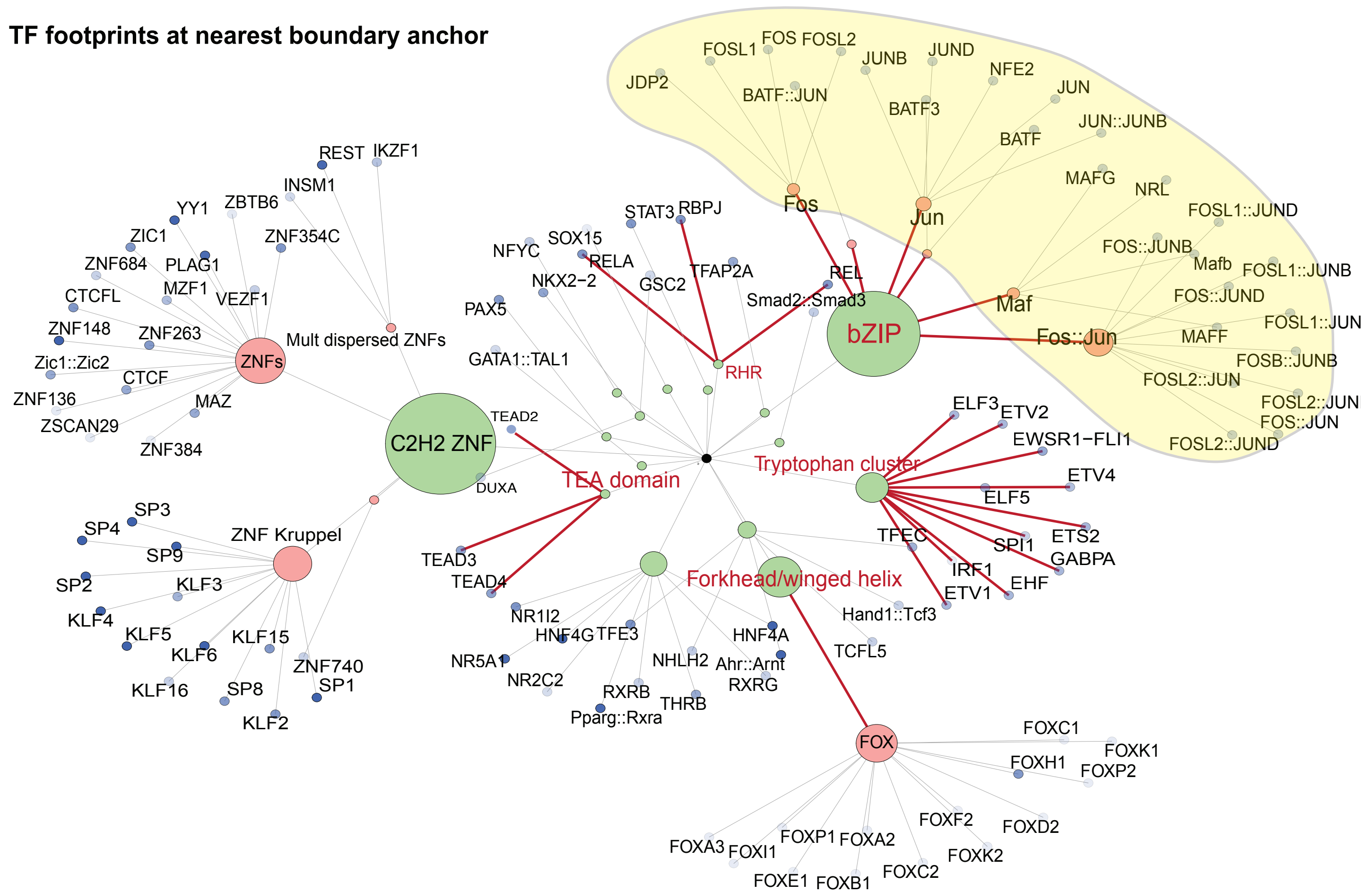

Supplementary Figure S7 (Figure S7)

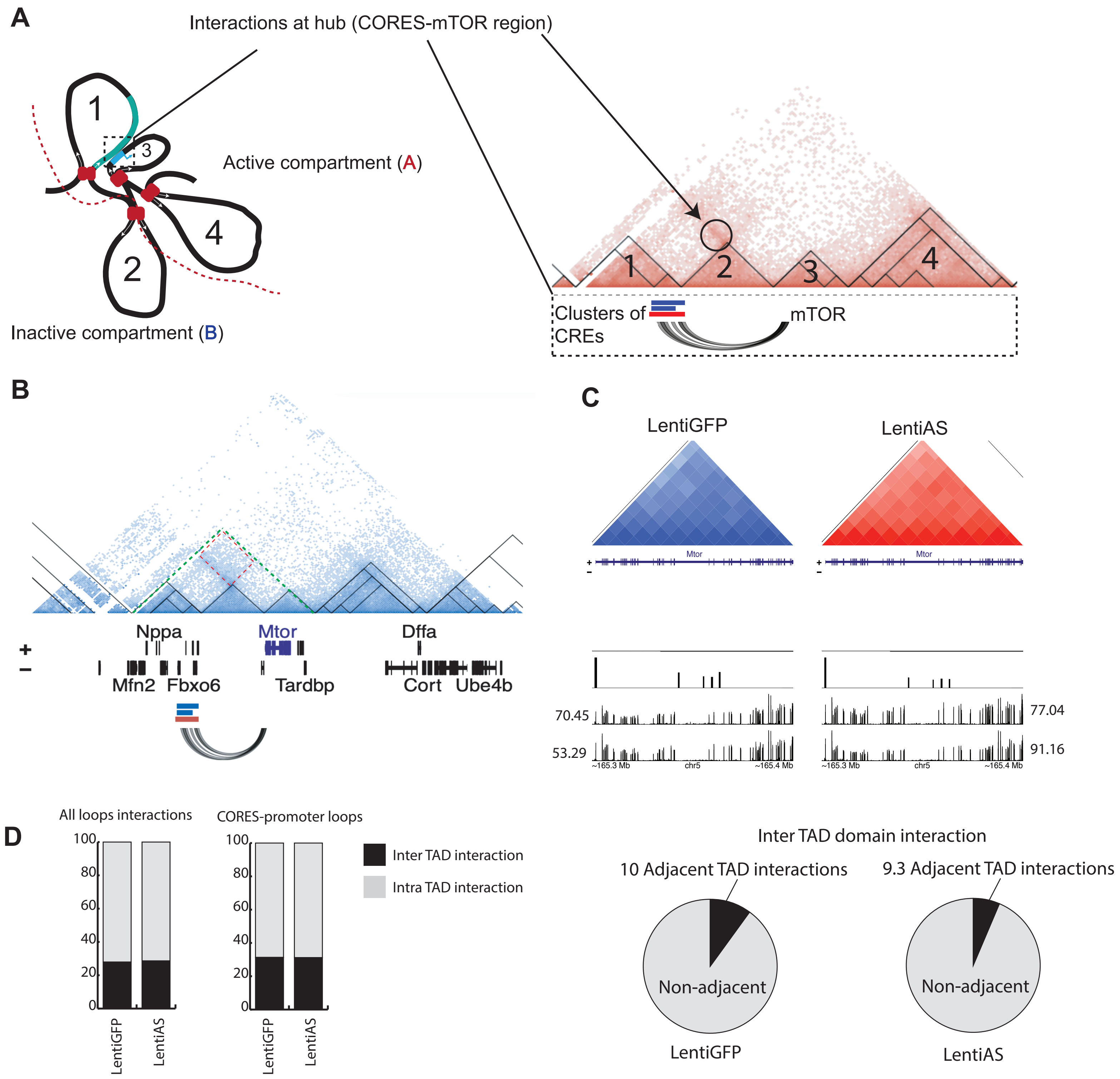

Supplement: Supplementary file 1 [file biomedicines-12-02594-s001.zip › biomedicines-3290204-supplementary-figure.pdf]
