# Supplementary material for: Role of the Egr2 Promoter Antisense RNA in Modulating the Schwann Cell Chromatin Landscape
Source: Biomedicines. 2024 Nov 13;12(11):2594. doi: 10.3390/biomedicines12112594 (PMC11592338; doi:10.3390/biomedicines12112594)
Supplement: Supplementary file 1 [file biomedicines-12-02594-s001.zip › biomedicines-3290204-supplementary-table.pdf]

Supplemental Experimental Procedures

Isolation of primary rat Schwann cells.

Isolation of rat primary Schwann cells from the sciatic nerves of female and male postnatal day 2 (P2) pups was described previously (Einheber et al., 1993). All animal use protocols were approved by the Institutional Animal Care and Use Committee of the Weis Center for Research, Geisinger Clinic and from the Animal Care and Use Committee of Rhode Island Hospital. All methods for animal use are reported according to the ARRIVE Essential 10 guidelines.

Rat Schwann cell culture

Rat Schwann cells were cultured as described earlier (Martinez-Moreno *et al*., 2017). Briefly, cells were cultured in DMEM supplemented with 10% FBS, 4 μM forskolin, 5 ng/ml heregulin-β1 and antibiotics, in surface modified 75cc *Primaria* flasks. Cells were fed every other day and passaged at 80% confluence.

Transfection experiments

Transfection of RSC with the target vectors/siRNAs or the negative controls were performed with the TransIT-X2 transfection reagent. Briefly, we plated $2x10^5$ cells per well in a 6-well plate dish (*Primaria*, Corning). Then we directly applied the TransIT-X2 in Opti-MEM containing 1 microgram of each vector/25nM of each siRNA. Overexpression of the Egr2-AS-RNA (and the backbone virus control carrying GFP) was performed with a lentiviral construct at a concentration of 2UFC/cell, with polybrene at a 1:1500 concentration, for 48 hours. GapMer against the Egr2-AS-RNA were added after 24 hours. The virus and GapMer sequences and generation were described in detail in previous work (Martinez-Moreno et al., 2017). GapMer sequence is proprietary of Exiqon (currently Qiagen). The plasmid to generate the lentivirus was deposited to Addgene and is available to order (#177737).

RT-PCR and qPCR

RNA was extracted from the cells, the sciatic nerves or the RIP eluates using Trizol and the PureLink RNA Mini Kit according to the manufacturer's protocol, following a DNase treatment. 300 ng of RNA was reversed-transcribed to cDNA using SuperScript III First Strand Synthesis System. For all qPCRs reported in the paper, we performed a no reverse transcription (RT) control amplification to verify the absence of genomic DNA contamination with GAPDH primers. For the qPCR, we obtained the cDNA as explained above, and we ran a qPCR using SYBR Green PCR Master Mix with the same PCR primers at a final concentration of 250 nM. Relative mRNA levels were normalized to GADPH and quantified using the comparative Ct method, and fold change was calculated compared to each control. All primer sequences are shown in Table 1.

Chromatin Accessibility Assay

Differential accessibility following Egr2-AS-RNA overexpression was assessed using the Chromatin Accessibility Assay kit (Cat #ab185901, Abcam plc, Cambridge, UK), according to the manufacturer's instructions. Briefly, chromatin was isolated from 1 million cells per group and subjected to nuclease digestion, which digests all accessible DNA, with a control group left undigested. After DNA precipitation, qPCR was performed on the promoters of interest (sequences in Table 1). Results were calculated as the ratio of amplification efficiency of the nuclease-treated sample to the non-digested control, with higher percentages indicating more open chromatin in the targeted promoter region.

c-Jun transcriptional activity TransAM AP-1 c-Jun

We used the Nuclear Extract Kit and the TransAM AP-1 c-Jun kit (Cat #46096, Active Motif, Carlsbad, CA). Briefly, we overexpressed Egr2-AS-RNA in RSCs as explained above and used 20 µg of the nuclear extract to run the transcription factor activity assay following the manufacturer's recommendations.

Supplementary Tables

Supplementary Table S1. Sequences

Application	Type	Sequence (5′-3′)	Modification
Egr2-AS inhibition	AS-RNA GapmeR	CCACCGTGTAATTCA	5' 6-FAM (Fluorescein)
RT-PCR	Egr2-AS-RNA -965FW primer	GTCAAGCTTCCATCTGGTC	None
qPCR	Egr2-AS-RNA -741FW primer	ACAAACAAACAGCCCAGACC	None
qPCR	Egr2-AS-RNA -317RV primer	AAGTCTCGGAACCGGAAT	None
qPCR	Rat GAPDH FW primer	CAACTCCCTCAAGATTGTCAGCAA	None
qPCR	Rat GAPDH RV primer	GGCATGGACTGTGGTCATGA	None
qPCR	Rat Egr2 FW primer	TCTTTCCGCTGTCCTCGAT	None
qPCR	Rat Egr2 RV primer	TGCTAGCCCTTTCCGTTGA	None
qPCR	Rat cJun FW primer	GCCTGCCTCTCTCAACTATGTA	None
qPCR	Rat cJun RV primer	TAGGACACCCAAACAAACAAAC	None
qPCR	Rat cJun promoter FW primer	CCCAAGACCTGTGTGAGAAT	None
qPCR	Rat cJun promoter RV primer	CTCACAGTTTGATTGGCTGAAA	None
RT-PCR/qPCR	FW primer 128 pCMV-HA-N Vector	GATCCGGTACTAGAGGAACTGAAAAAC	None
RT-PCR/qPCR	RV primer 32 pCMV-HA-N Vector	GTGGTTTGTCCAAACTCATC	None

ATACseq	Universal primer	AATGATACGGCGACCACCGAGATCTAC ACTCGTCGGCAGCGTCAGATGT*G	5′-Phosphate Internal-Phosphorothioate
ATACseq	Indexing primer #1	CAAGCAGAAGACGGCATACGAGATTCG CCTTAGTCTCGTGGGCTCGGAGATG*T	5′-Phosphate Internal-Phosphorothioate
ATACseq	Indexing primer #2	CAAGCAGAAGACGGCATACGAGATCTA GTACGGTCTCGTGGGCTCGGAGATG*T	5′-Phosphate Internal-Phosphorothioate
ATACseq	Indexing primer #3	CAAGCAGAAGACGGCATACGAGATTTC TGCCTGTCTCGTGGGCTCGGAGATG*T	5′-Phosphate Internal-Phosphorothioate
ATACseq	Indexing primer #4	CAAGCAGAAGACGGCATACGAGATGCT CAGGAGTCTCGTGGGCTCGGAGATG*T	5′-Phosphate Internal-Phosphorothioate

Supplementary Table S2. Reagents used in this manuscript.

Reagent or Resource	Application	Source	Identifier
**Oligonucleotides – See Supplementary Table**			
**Antibodies**			
B-Actin (8H10D10) Mouse mAb (HRP Conjugate)	Western Blot	Cell signaling	12262
WDR5 (D9E1I) Rabbit mAb	IP, RIP, Western Blot	Cell signaling	13105S
Ezh2 (D2C9) XP® Rabbit mAb	IP, RIP	Cell signaling	5246
Histone H3K4Me3 antibody (mAb)	ChIP	Active Motif	61379
ChIPAb+ Trimethyl-Histone H3 (Lys27)	ChIP	Millipore-Sigma	17-622
Mediator 1 (MED1) antibody	RIP	Cell signaling	51613
Rabbit IgG, polyclonal - Isotype Control (ChIP Grade)	IP, RIP, ChIP	Abcam	ab171870
Anti-Egr2 antibody [EPR4004]	Western Blot	Abcam	ab108399
Recombinant Anti-KMT6/EZH2 antibody	Western Blot	Abcam	ab191080
**Chemicals, Peptides, and Recombinant Proteins**			
DMEM, high glucose, pyruvate	Cell culture	Gibco	11995040
FBS Benchmark USDA	Cell culture	Gemini Bio	100106
Forskolin	Cell culture	Sigma-Aldrich	F3917
Recombinant Human NRG1-beta 1/HRG1-beta 1 EGF Domain, CF	Cell culture	R&D Systems	396-HB/CF

TransIT X2 Transfection Reagent	Transfection	MirusBio	MIR6003
Opti-MEM Reduced serum media	Transfection	ThermoFisher	31985062
Polybrene Infection / Transfection Reagent	Cell culture	Sigma-Aldrich	TR-1003
RIPA buffer	IP and co-IP	Santa Cruz Biotechnology	24948
Protein A/G PLUS-Agarose	IP and co-IP	Santa Cruz Biotechnology	2003
TRIzol Reagent	RNA extraction	Invitrogen	15596026
**Commercial Assays and Kits**			
RNA ChIP-IT	RIP	Active Motif	53024
ChIP-IT High Sensitivity ®	ChIP	Active Motif	53040
PureLink RNA Mini Kit	RNA extraction	Ambion	12183018A
Immunoprecipitation Kit: for DYKDDDDK (FLAG®)	Immunoprecipitation	Rockland Immunochemicals	KBA-319-383
Nuclear extract kit	Transcription Factor ELISA	Active Motif	40410
TransAM AP-1 c-Jun kit	Transcription Factor ELISA	Active Motif	46096
In-Fusion® HD Cloning Plus	Cloning	Takara Bio	638920
Chromatin Accessibility Assay kit	Chromatin accessibility	Abcam	185901
Nextera DNA Library Prep Kit	ATACseq	Illumina	FC-121-1031
MinElute Reaction Cleanup Kit	ATACseq	QIAGEN	28204

KAPA Library Quantification Kit for Illumina Platforms	ATACseq	KAPA Biosystems	KK4824
Arima-HiC kit	HiC	Arima Genomics	A510008
KAPA Hyper Prep Kit to generate libraries	HiC	KAPA Biosystems	KK8500
SuperScript III First-Strand Synthesis System	RT-PCR	ThermoFisher	18080400
SYBR Green PCR Master Mix	qPCR	Applied Biosystems	4309155
NEBNext High-Fidelity 2X PCR Master Mix	ATACseq	New England BioLabs	M0541
	**Recombinant DNA and RNA Products**		
pLVX-Egr2ASRNA	Transfection	Addgene	177737
ON-TARGETplus Rat Egr2 siRNA	Transfection	Dharmacon	L-096344-02-005
ON-TARGETplus Non-targeting Control siRNA	Transfection	Dharmacon	D-001810-01-05
pCDNA3 Flag Wdr5 expression plasmid	Transfection	Addgene	15552
1436 pCDNA3 Flag HA (empty backbone) plasmid	Transfection	Addgene	10792
pCMV-HA-N	Cloning and transfection	Takara Bio	631604
TruSeq DNA Single Indexes Set A	Library preparation	Illumina	20015960
	**Plates**		
Primaria™ 75cm² flasks	Cell culture	Corning	353810
Primaria™ 6-well Cell Clear Flat Bottom Surface-Modified	Cell culture	Corning	353846

Instruments		
**Model**	**Application**	**Company**
Homogenizer Pro 200	Tissue homogenization	Proscientific Inc
Misonix Sonicator 3000	Sonication ChIP, RIP	Cole Palmer
ABI StepOne Plus instrument	qPCR	Applied Biosystems
s220 instrument	Sonication Hi-C	Covaris
GloMax® Discover Microplate Reader	Luminescence	Promega

Supplemental Figure legends

Figure S1: Validation experiments

A) Expression of the EGR2-AS in Schwann cells results in increased protein expression of C-JUN and inhibition of the EGR2 protein expression. Significance was calculated with a Student's t-test (For EGR2, N = 8, 3 independent experiments, **p = 0.0017, dF = 7; For C-JUN, N = 10, 4 independent experiments, **p = 0.0016, dF = 18).

B) Egr2 siRNA (Pool of 4 independent siRNAs) induces significant inhibition of Egr2 protein expression but does not affect c-Jun expression. P<0.005, Student's t-test (n=4). C) Western blots of EZH2 and WDR5 following chromatin immunoprecipitation with EZH2 and WDR5 antibodies respectively, shows equal presence of EZH2 and WDR5 in the IP lysates from Lenti-GFP and Lenti-AS samples. D) Expression of EGR2-AS and its inhibition by an oligonucleotide GapmeR. The results are plotted as Fold Change (or 2DDCt) of the experimental condition (Lenti-AS and Lenti-AS + GapmeR) compared to the control (Lenti-GFP). An average of 5 different experiments was calculated and plotted, and significance was calculated with one-way ANOVA (F (2,11) = 2.537, p < 0.0001) followed by independent t-tests (LentiAS vs LentiGFP, **** p < 0.0001; LentiAS + Gapmer vs LentiGFP, ns; LentiAS vs LentiAS + Gapmer, *** p = 0.0004).

Figure S2: Expression of the EGR2-AS induces changes in chromatin accessibility A) Similarity matrix analysis is shown as a heat map and the intensity of the color indicates cross-correlation between the compared groups. B) Heatmap of differentially accessible genes (defined as fold change > 1.5, with a P-value < 0.05). C) IGV of the mapped region relative to the c-Jun gene, shows more accessibility on the promoter on the LentiAS groups compared to LentiGFP. The position coordinates on the chromosome 5 is marked on the below line. The position and depth of mapped reads with the range are indicated at the left. D) Transcriptional activity of c-Jun was assessed in Schwann cells after overexpression of Egr2-AS using a TransAM reporter assay. Data

are presented as mean ± SEM from three to five independent experiments per group. Statistical significance was determined using a Student's t-test, with *$p < 0.05$ indicating significant upregulation of c-Jun activity in the Egr2-AS overexpression group compared to the control group Lenti-GFP. This experiment also shows that the inhibition of Egr2 doesn't increase c-Jun activity. E)

Figure S3: Loops description
A) Total number of loops per sample. B) Size of loops per sample. C) An example of significant loops detected in each condition. D) Gained and lost loops per chromosome.

Figure S4: Characterization of COREs

A) Shows total number of COREs. B) Density plot of TF occupancy of all COREs across all samples. C) COREs-promoter length between the AS-RNA expressing cells and GFP controls. D) QC showing equal distribution of library fragments between Lenti-GFP and Lenti-AS.

Figure S5: Characterization of loop interaction between mTOR and COREs A) Depiction of TADs harboring mTOR and COREs in the AS-RNA and control samples. Interdomain interaction shown here as area of increased interaction intensity relative to surrounding bins. B) Total footprint detected at all loop anchors and shown by distinct loop anchors. Loop anchors named based on distance from nearest TAD boundary. C) Hi-C interaction map emphasizing interdomain mTOR loop interactions. D) QC for HiC replicates of Lenti-GFP and Lenti-AS.

Figure S6: Network of TF binding dynamics at the mTOR interacting regulatory element/super enhancer.

Network of TF binding at the loop interacting with the COREs at the nearest TAD boundary. Maroon color emphasizes TF class and families that experience a statistically significant increase in TF binding following expression of the EGR2-AS (Wilcoxon test, p-val <0.05).

Figure S7: Characterization of mTOR interaction hub

A) Relates TAD interacting model to Hi-C data. B) MetaTAD region is emphasized with a green dotted line, while red box shows interdomain interaction. C) RNA-seq signal with normalized reads at the mTOR harboring TAD. D) Ratio of inter and intra TAD interactions between AS-RNA expressing cells and controls.
